# Percutaneous removal of sacroiliac screw following iatrogenic neurologic injury in posterior pelvic ring injury: A case report

**DOI:** 10.1016/j.ijscr.2020.01.004

**Published:** 2020-01-14

**Authors:** Jawaher Mohammed Alkhateeb, Sabrina Saphia Chelli, Abdulla Anwar Aljawder

**Affiliations:** a Royal College of Surgeons in Ireland, Medical University of Bahrain, Busaiteen, Bahrain; b Department of Orthopedic Surgery, Bahrain Defense Force Hospital, Riffa, Bahrain

**Keywords:** Percutaneous sacroiliac screw, Neurologic injury, Pelvic ring injury, Sacral fracture, Implant removal, Case report

## Abstract

**Introduction:**

Percutaneous sacroiliac fixation is an effective minimally invasive method for posterior pelvic ring stabilization. Screw misplacement, and subsequent neurologic injury are two well described complications. Managing those complications however is under-reported.

**Case:**

A young female, sustained an unstable pelvic ring injury as a victim of motor vehicle collision. Following percutaneous sacroiliac screw fixation, she complained of L5 nerve root radiculopathy, and muscle weakness. Percutaneous removal of the screw after a wait period for fracture union resulted in immediate symptoms relief.

**Discussion:**

Safe sacroiliac screw placement is technically demanding requiring good understanding of sacral complex morphology and its anatomic variants. Risk of screw misplacement, and potential neurologic injury increases in dysmorphic sacra, or with inaccurate fracture reduction. Advances in intraoperative imaging modalities have been introduced in an attempt to improve accurate screw insertion. Literature is scarce with reports discussing removal of sacroiliac screw. Technique of screw retrieval is also controversial.

**Conclusion:**

This case addresses management of an iatrogenic neurologic complication following percutaneous sacroiliac screw fixation. Our experience showed that, percutaneous retrieval of an intact misplaced sacroiliac screw is achievable, resulting in complete resolution of neurologic symptoms.

## Highlights

• Percutaneous sacroiliac (SI) screw fixation is a well-known method for fixing posterior pelvic ring injuries.
• An understanding of the anatomical variations of sacral morphology, and proper reduction are both mandatory for accurate screw placement.
• Despite the introduction of intraoperative navigation, SI screw misplacement still occurs.
• The optimal technique for (SI) screw removal is controversial.
• Percutaneous extraction of an intact SI screw resulted in complete neurologic recovery.

## 1 Introduction

There is a rising incidence of severe pelvic injury following motor vehicle collusion, constituting a major cause of morbidity and mortality [1]. Unstable pelvic ring disruption involving the posterior ring accounts for 40% of cases [1]. Injuries include; sacroiliac joint dislocation, or fracture- dislocation, iliac wing fractures, and sacral fractures. While isolated un-displaced sacral fractures are generally treated non-operatively, it is essential to accurately reduce and stabilize displaced fractures for better functional results [[2], [3], [4]].

Posterior pelvic ring fixation has been achieved using variable methods; each has its own advantages and drawbacks [5,6]. The most commonly used is the percutaneous sacroiliac (SI) screw fixation; it involves inserting up to 80 mm cannulated screw from the lateral ilium traversing the SI joint and into the upper sacral vertebral body after obtaining accurate reduction. It can be inserted in prone, supine, or lateral decubitus position [5,6]. The goal is to achieve stable fixation to allow for bone growth and subsequently fusion of SI joint. This procedure gained popularity due to its advantages of minimal soft tissue disruption, negligible blood loss, reduced operative time, and minimal implant prominence compared to open methods [2,3,5,6]. However, placement of the SI screw is technically difficult requiring good understanding, and three-dimension imagination of sacral morphology and its anatomical variance [3,7]. To facilitate accurate visualization of the sacrum, many imaging modalities have been utilized intraoperatively; fluoroscopy, computed tomography, and intraoperative navigation [8].

Accurate reduction is necessary for safe SI screw insertion. Different clinical, radiographic, and anatomical studies repeatedly described the potential danger of injuring nearby neurovascular structures [[9], [10], [11], [12]]. SI screw misplacement is frequently observed in 3–29%, and it is associated with L5 nerve root injury in 0.5–8% of reported cases [7]. Several studies attempted to improve accuracy of SI screw placement in order to avoid subsequent potential complications [13,14]. However, only few tried to address managing an iatrogenic neurologic injury following a misplaced SI screw [15,16].

We present our experience in managing neurologic complication following percutaneous SI screw fixation of sacral fracture in a young female patient. Immediately following the removal of SI screw, our patient had complete resolution of neurologic symptoms.

This case report was written according to the SCARE criteria [17].

## 2 Case presentation

**Fig. 1:**
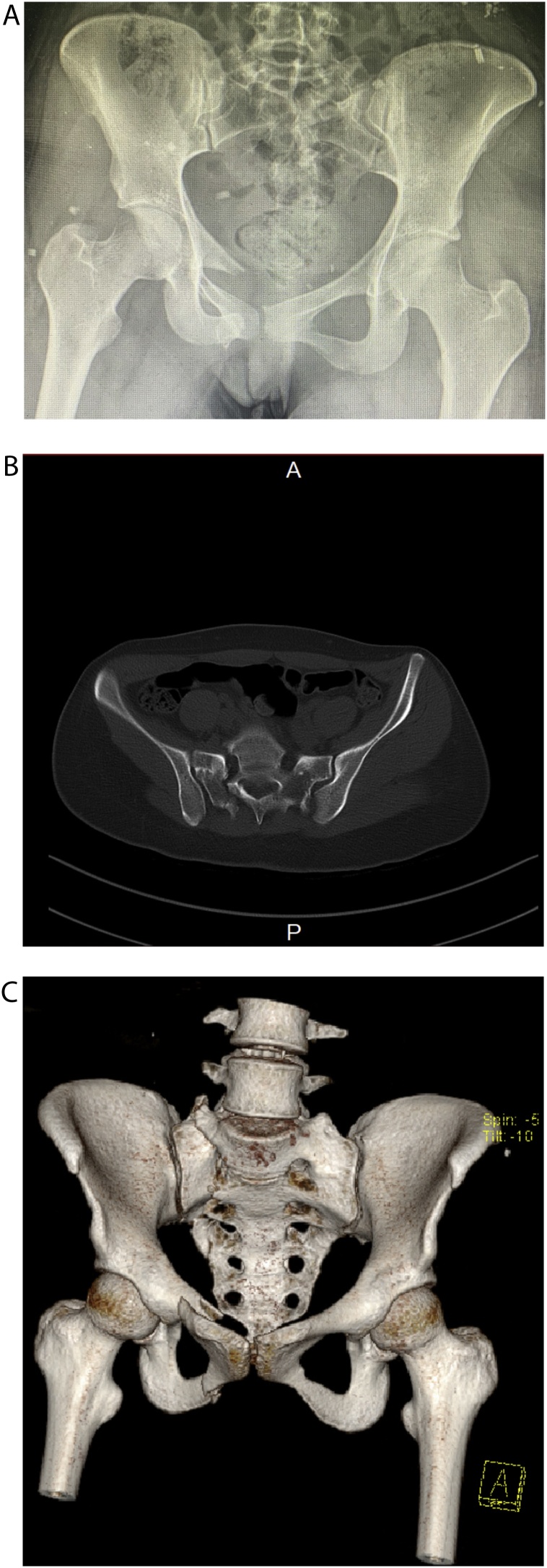
A: Anteroposterior pelvis view shows suspicious vertical displacement of right hemipelvis due to right sacral fracture, and right anterior ramus fracture. B: CT (axial cut), and C (3D reconstruction image); delineates clearly that the fracture is transforaminal, and is displaced posteriorly, no vertical displacement is identified based on CT images.

A 20-year-old female patient was brought to our hospital by ambulance following motor vehicle accident. She sustained a rotationally and vertically unstable pelvic ring injury, involving fracture of the right superior pubic ramus with an ipsilateral transforaminal sacral fracture (Fig. 1). Distal neurovascular examination was normal. The patient is not known to have any medical or psychological illness and had no surgical procedures in the past.

**Fig. 2:**
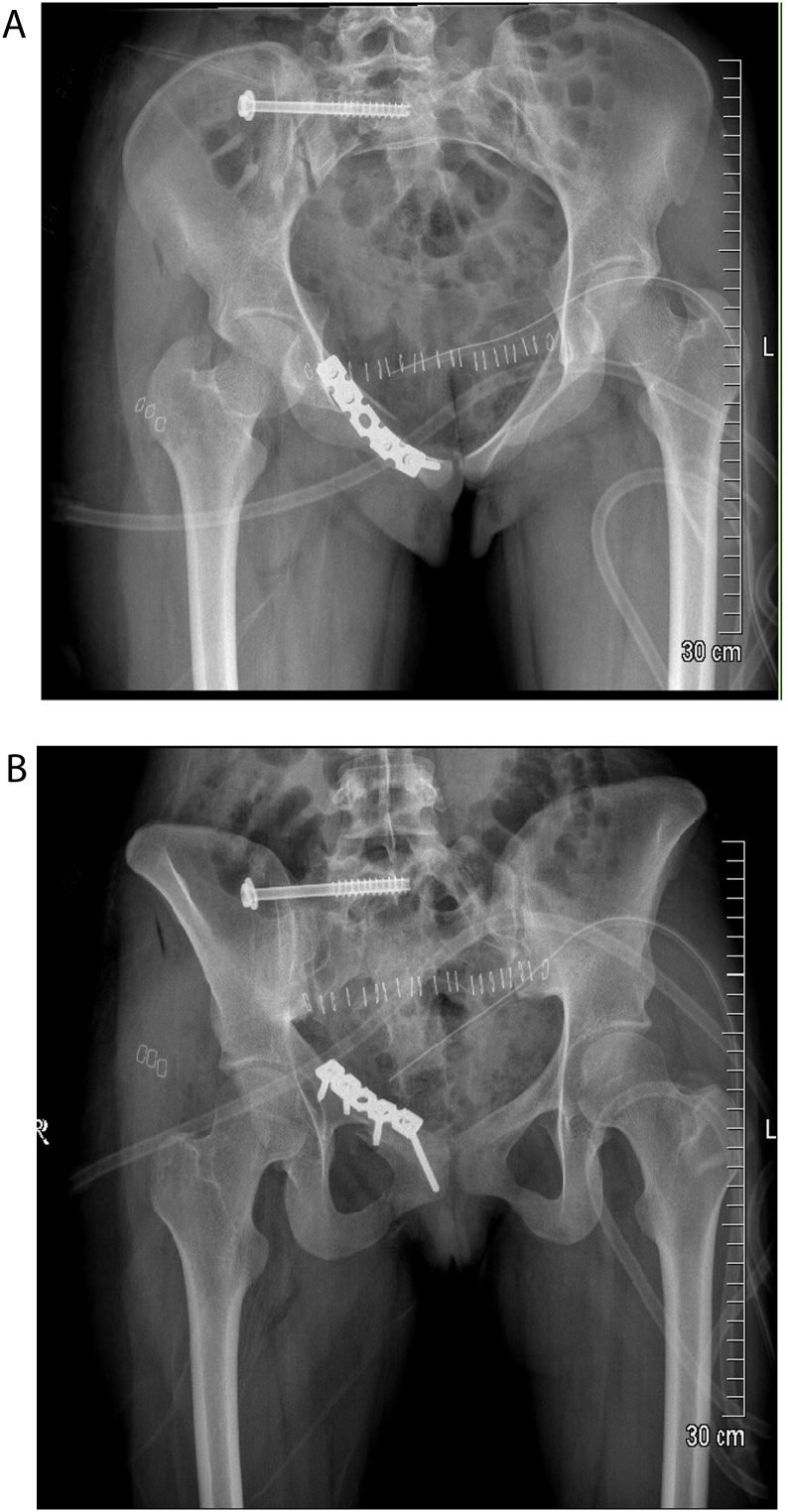
Images (A: inlet), and (B: outlet views) shows the pelvic ring fixation with pubic plate anteriorly, and sacroiliac screw posteriorly.

The patient was taken to the operating theater within 48 h for surgical fixation. Procedure was performed by a senior orthopedic surgeon specialized in trauma of lower extremities. Under general anesthesia, she was placed supine on radiolucent table. Open reduction and internal fixation of the superior pubic ramus fracture with plate and screws was done using an anterior horizontal Pfannenstiel approach. That was followed by percutaneous sacroiliac (SI) screw fixation of the ipsilateral sacral fracture. Using standard fluoroscopic imaging, iliac cortical density, frontal edge of the sacral canal, and sacral promontory landmarks were identified. Percutaneous reduction was then obtained. After demarcating the S1/S2 safe area for percutaneous SI screw, a guide-wire was inserted into the posterior cortex of the S1 vertebral body using alternating inlet and outlet views to ensure proper trajectory of the guidewire. 7.3 mm cannulated screw with washer was inserted obliquely across the SI joint, supero-anterior to the sacral ala. Correct positioning of the cannulated screw was confirmed via inlet, outlet, and lateral views (Fig. 2).

**Fig. 3:**
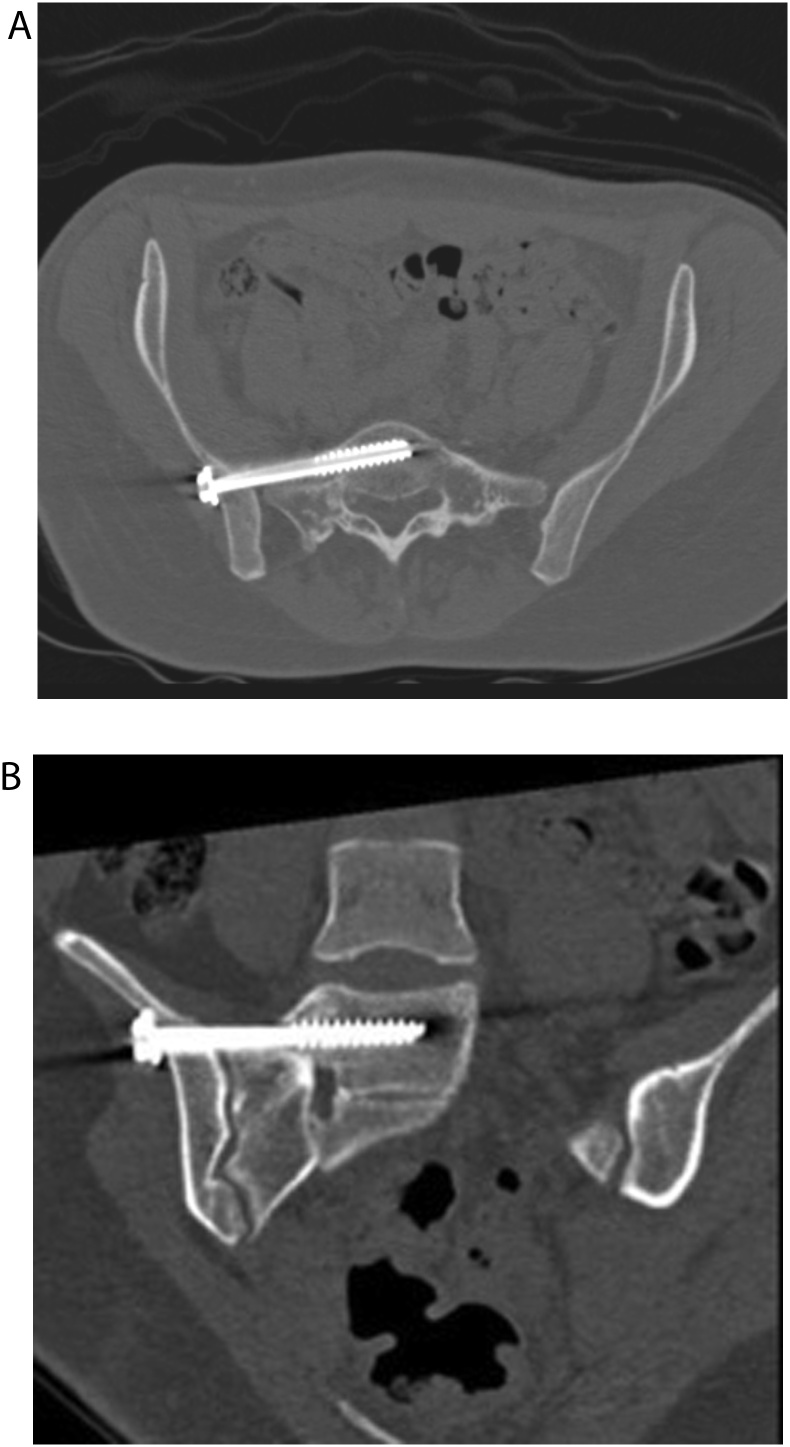
CT: (A: axial cut), and (B: coronal cut) shows an extraosseous portion of the screw perforating anterior cortex of sacral ala.

Post operatively, the patient complained of L5 radicular pain starting at her right buttock and radiating to her posterior thigh and leg. It was also accompanied by motor weakness; 4/5 in both tibialis anterior and extensor hallucis longus, and hyperesthesia along L5 dermatome. Initially, symptoms were thought to be attributed to nerve irritation following intraoperative fracture manipulation, and therefore, patient was assured and discharged home with analgesics after non-weight bearing mobilization. Upon her two-week follow up, patient symptoms persisted. Computer Tomography (CT) scan of the pelvis was then obtained and revealed that the SI screw was breaching the anterior cortex of first sacral body (Fig. 3). Decision was made to attempt percutaneous removal of the SI screw after a wait period of 5 weeks to allow for callus formation at the sacral fracture site, thus ensuring stability of the posterior pelvic ring.

Removal was straightforward; patient was placed supine on a radiolucent table. Using standard fluoroscopic views, a stab incision was made over the S1 screw site. A guide wire was then advanced to the central aspect of the cannulated screw. Securing a cannulated screwdriver to the screw head was insured confirming its position via inlet and outlet views, and reversal extraction was achieved taking care not to lose its washer.

**Fig. 4:**
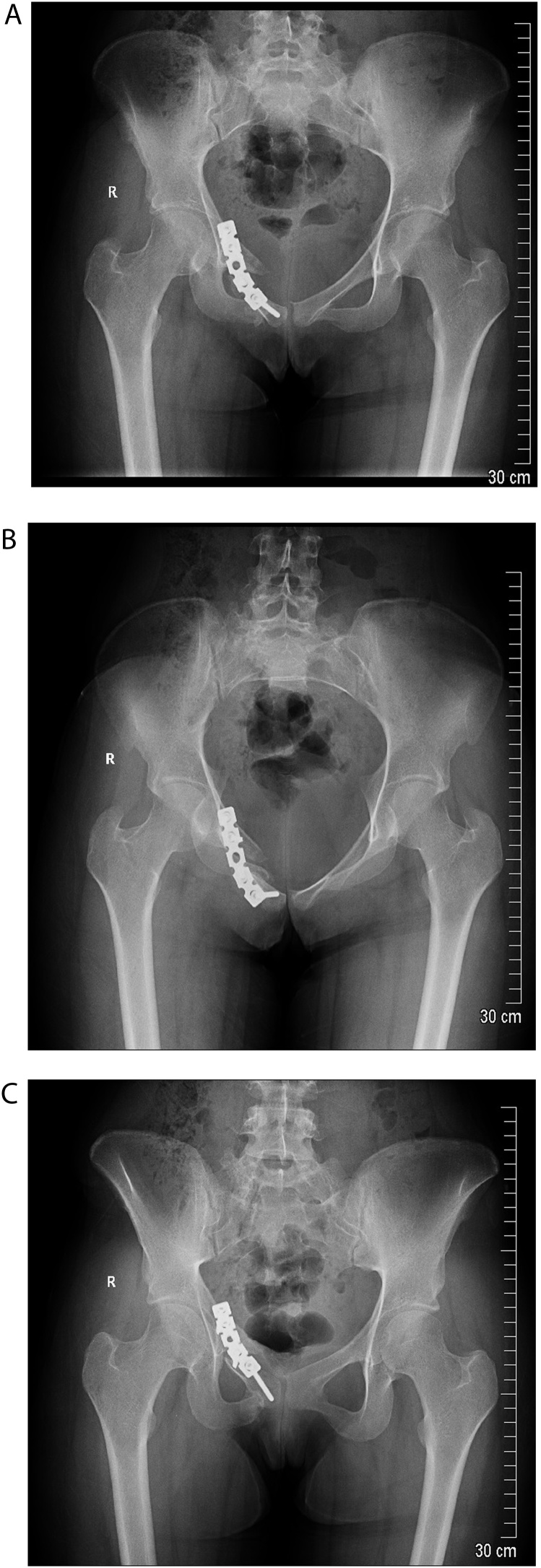
Late follow up images after removal of the SI screw shows well reduced satisfactory union at the sacral fracture site (A: AP), (B: inlet) (C: outlet) views.

Subsequent to removal, patient’s symptoms improved immediately with complete relief of radicular symptoms as well as restoration of ankle and toes dorsiflexion power. The patient was compliant to our mobilization protocol, which started with toe touch to partial weight mobilization for three months, and only until late follow up radiograph was satisfactory (Fig. 4), she started to weight bear as tolerated. In regard to functional outcomes, patient is pain-free and has returned to all her activities.

## 3 Discussion

Percutaneous SI screw fixation is proven to be both; safe and effective minimally invasive method for stabilization of posterior pelvic ring injuries [2,3,[5], [6], [7]]. Aberrant insertion is a common complication that would potentially damage nearby neurologic structures [2,3,[9], [10], [11], [12]]. This danger is explained by the intimate relation between L5 nerve root and the anterior cortex of sacral ala, estimated in several anatomic studies by a distance as close as to 1 mm [10,10,11,12]. As little as 4 mm of misdirection can misplace the screw into the S1 foramina or perforate through the anterior sacral cortex [5]. Centrally placed SI screw in the first sacral body ensures safe osseous corridor of 7 mm distance away from neurovascular structures. This zone is diminished in either fracture displacement, or dysmorphic sacra [11]. Displacement of transforaminal sacral fracture reduces the opposition between central and lateral fragments [18].

While safe placement is technically demanding due to sacral complex anatomy, it is more challenging in dysmorphic sacra [19]. Wide variation of sacral morphology exists in up to 40% of general population, all of which does not allow for safe passage of trans-osseous screw placement [14,20]. Consequently, misplaced instrumentation can occur in 29% of dysmorphic sacra, compared to 12% in sacra without dysmorphism [7]. The introduction of intraoperative navigation allowed surgeons to accurately visualize the sacrum, and to immediately assess screw trajectory. In a recent systematic review [7], CT navigation was found to be the modality with the lowest malposition rate (0.1%). However, aberrant insertion still occurs in dysmorphic sacra with no significant difference in risk of cortical breach between standard imaging and navigation [14].

Despite the common incidence of malposition and subsequent neurologic injury, only few reports address its management. We found two cases described managing a misplaced screw by its removal. In both cases, screw was either bent or broken. In addition, technique of screw retrieval was controversial.

Weil et al. [15] reported a patient suffering from L5 neurologic injury, presenting three months following SI joint fixation. The SI joint was mal-reduced, with mispositioned, and bent screw. Using intraoperative neurologic monitoring, they described an open removal of SI screw with L5 nerve root exploration, followed by SI joint fusion. They emphasized on the importance of extensile approach, assuming that blind screw extraction would result in further neurologic damage. Eldafrawy et al. [16] described a novel technique to extract the SI screw percutaneously by pushing the screw from the contralateral side. Their technique worked successfully in removing a broken screw in one case. It was aborted in another case, with conversion to open removal after multiple failed attempts.

In our patient, the SI joint was well reduced necessitating no salvage procedure. A decision was made to wait for callus formation at the sacral fracture site, ensuring posterior pelvic ring stability, before any removal attempts. We were convinced that, because the screw was intact, and the screw tip is intraosseous, a percutaneous extraction would be amenable. If the scenario was different, and the screw was either broken, bent, or the tip was extraosseous, we would have been very hesitant to attempt a percutaneous retrieval, and probably would have leaned toward favoring an open exploration.

## 4 Conclusion

Percutaneous SI screw fixation is a well-known effective method for treating posterior pelvic ring instability [2]. Complications involving screw misplacement and neurologic injury are well documented [3]. Accurate reduction, careful understanding of sacral anatomy, and the use of proper imaging technique are all essential to ensure proper SI screw placement [14]. In an unwanted scenario of misplaced screw, and subsequent neurologic injury, we recommend attempting percutaneous removal, given that the screw is intact, and the SI joint is well reduced.

## Sources of funding

None.

## Ethical approval

Ethical approval was obtained from the Institutional Review Board at the Royal Medical Services of the Bahrain Defence Force (file attached).

## Consent

Written consent was taken from the involved patient for the presentation of the case as well as corresponding radiography for the manuscript (available upon request).

## Author contribution

Jawaher Alkhateeb: collecting data, literature review, reviewing and editing the paper.

Sabrina Chelli: collecting data, literature review, writing and submission of the paper.

Abdulla Aljawder: supervising, collecting data, reviewing and editing the paper.

## Registration of research studies

researchregistry5184.

## Guarantor

Abdulla Aljawder (supervisor of this case report).

## Provenance and peer review

Not commissioned, externally peer-reviewed.

## Declaration of Competing Interest

No conflicts of interest.
